# Nocturnal Homing: Learning Walks in a Wandering Spider?

**DOI:** 10.1371/journal.pone.0049263

**Published:** 2012-11-07

**Authors:** Thomas Nørgaard, Yakir L. Gagnon, Eric J. Warrant

**Affiliations:** 1 Biology Department, Lund University, Lund, Sweden; 2 Biology Department, Duke University, Durham, North Carolina, United States of America; Utrecht University, The Netherlands

## Abstract

Homing by the nocturnal Namib Desert spider *Leucorchestris arenicola* (Araneae: Sparassidae) is comparable to homing in diurnal bees, wasps and ants in terms of path length and layout. The spiders' homing is based on vision but their basic navigational strategy is unclear. Diurnal homing insects use memorised views of their home in snapshot matching strategies. The insects learn the visual scenery identifying their nest location during learning flights (e.g. bees and wasps) or walks (ants). These learning flights and walks are stereotyped movement patterns clearly different from other movement behaviours. If the visual homing of *L. arenicola* is also based on an image matching strategy they are likely to exhibit learning walks similar to diurnal insects. To explore this possibility we recorded departures of spiders from a new burrow in an unfamiliar area with infrared cameras and analysed their paths using computer tracking techniques. We found that *L. arenicola* performs distinct stereotyped movement patterns during the first part of their departures in an unfamiliar area and that they seem to learn the appearance of their home during these movement patterns. We conclude that the spiders perform learning walks and this strongly suggests that *L. arenicola* uses a visual memory of the burrow location when homing.

## Introduction

**Figure 1:**
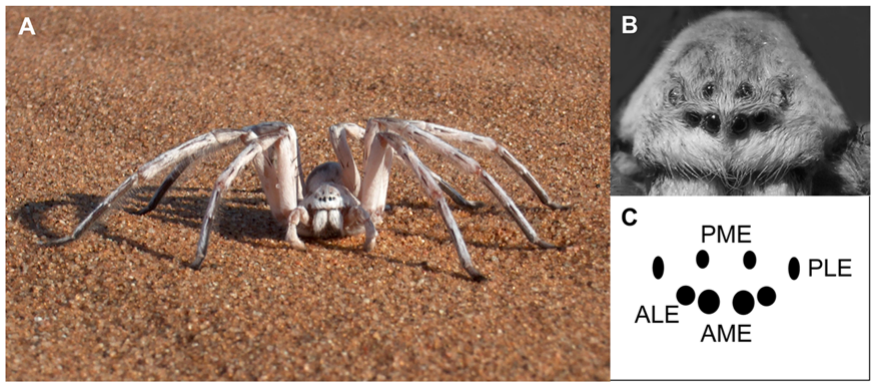
The navigator. **A.** Adult male *L. arenicola*. **B.** Close-up view of the eye arrangement on the carapace of *L. arenicola*. **C.** Schematic representation of the position of the anterior lateral eyes (ALE), anterior median eyes (AME), posterior median eyes (PME), and posterior lateral eyes (PLE). (**B** and **C** modified from Nørgaard et al. 2008).

The use of spatial characteristics in a visual scene to identify goal locations is widespread in arthropods [1]–[5]. In the Namib Desert, the wandering spider *Leucorchestris arenicola* (Araneae: Sparassidae) (Fig. 1) depends on visual cues when navigating back to its home burrow [6], despite being nocturnal and mostly active on moonless nights [7]. The spider thus uses vision even under very dim light conditions. The burrow is superbly camouflaged and beyond a few centimetres it is not visible to the spider [8], [9] but if there are prominent nearby landmarks it is able utilise these to find the hidden burrow even after being passively displaced several meters inside an opaque box [10]. However, most often there are no large local landmarks in the spider's habitat and it is currently unclear what visual information it then collects and how this information is applied when navigating. The adult male spiders regularly set out to search for females – at times on long excursions stretching hundreds of meters away from their burrows [11]–[13]. They nevertheless return to their burrows with minimal retracing of the outward path [8]. These nocturnal excursions are comparable to the foraging behaviour of diurnal bees, wasps and desert ants [4]. Path integration is the principal navigational system in the insects [14], [15], but it is unknown if this is also the case in the spiders. Bees and wasps leaving their nest for the first time perform learning (or “orientation”) flights: they turn in mid air to face their nest and fly backwards away from it through increasing arcs higher and higher above ground [3], [16]–[20]. Namib Desert ants perform conspicuous rotation movements if the visual surroundings of their nest change [21]. During these learning flights and walks the insects acquire knowledge of the visual scenery identifying the nest location by aligning themselves towards their goal and by viewing it from so-called inspection points at the end of the flight arcs [3], [22]–[24]. This behaviour is most pronounced in naïve individuals leaving a goal location for the first time [20]. The collected visual information is stored by the insects and appears to be recalled in “snapshot matching” navigational strategies where homing is achieved by comparing memorised images to the current visual input [5], [25]. Stereotypic movement patterns confined to the initial phase of departure appears to be an essential component in visual homing [3], [20], [26]–[28]. The presence of such movement patterns therefore makes it highly likely that the homing of the navigator in question is based on collection, storing and retrieving of visual information. If *L. arenicola* uses “snapshot matching” in a fashion similar to diurnal insects, then the spiders would be likely to show distinct movement patterns when departing, and this should be especially pronounced in naïve spiders (i.e. spiders leaving a burrow for the first time at a new and unfamiliar location).

We recorded and analysed the fine details of the spiders' movements near their burrows with infrared sensitive cameras and computer tracking techniques and found that *L. arenicola* do perform distinct sinusoidal movement patterns when departing. Based on how the spiders keep the burrow in line of sight along the sinusoidal paths, and how the paths change shape as the spiders gain experience, we conclude that these distinctive movement patterns are learning walks.

## Materials and Methods

The recordings of the spiders' movement paths were carried out in the Namib Desert (S 23 34.63, E 15 02.56). The experimental site had very few natural landmarks. The only prominent landscape feature is a dune ridge to the East. Spiders newly caught at sites several kilometres away were placed at different locations within the experimental site and induced to construct a burrow [10]. At the time of their first departure at the experimental site the spiders were therefore completely unfamiliar with the area. We have defined this as a naïve spider. Only one spider at a time was present at the experimental site during the experiments. An infrared-light-sensitive video camera (Sony HDR-Sr11e) fitted with a wide-angle lens (Kenko KCW-042) was placed on a tripod. The camera's field of view covered an area of two meter radius around the burrow. This area was illuminated with an infrared floodlight (LIR850-70, LDP LLC, Carlstadt, New Jersey, USA; peak wavelength  = 850 nm and 70° beam spread). The floodlight peak wavelength of 850 nm is well above the spiders' visual range: this peaks below 550 nm and is therefore invisible to the animals [6]. Recordings started at sunset and lasted for approximately five hours. This time span covers the time period in which the spiders are most likely to be active [7]. When conditions in the desert allowed, the spiders' foot prints in the sand were also observed [8]. All necessary permits for the described non-invasive field studies were obtained from the Gobabeb Training and Research Centre.

The recorded video sequences were processed using MATLAB (R2008b). Each video sequence was imported in batches (25 frames at a time) and converted from RGB (8 bit) to gray-scale. The frames in each batch were cropped into a 300*300 pixel frame, resulting in an intensity matrix of size 300*300*25 (the frame size of the batches was sometimes adjusted to accommodate noise and the spider's movement speed). The cropped area was continuously centred on the location of the spider in the video sequence. The intensity matrix was subtracted by its temporal mean, making the absolute values of invariant pixels small and maximizing the absolute values of variant pixels. A threshold of eight (about 3% of 256 intensity values) was applied to detect intensity difference across time in the frame-batch (this threshold was also adjusted for noise levels in some videos). All connected objects in the batch were then identified. Their volume and shape was used to determine which is most likely to be the spider. Following this all the videos were calibrated using the Camera Calibration Toolbox for MATLAB [29]. Each recording session started with filming a one by one meter checkerboard with nine by nine black and white squares (11.11×11.11 cm) placed on the ground directly above or close to the burrow. Using this method the deformation of the image caused by the position and viewing angle of the camera (as well as aberrations from the lens) was compensated for. The spider paths were spatially transformed in order to be viewed directly from above using the data from the camera calibration. The tracks were then manually cleaned from obvious mistakes by the tracking algorithm. These mistakes occurred only in the periphery of the scene, where the dimmer light conditions occasionally prevented the algorithm from precisely determining the spider's location. Spider positions were regarded as mistaken if they implied the spider repeatedly jumped several centimetres within less than three seconds. Finally the paths were smoothed using a Kalman filter in order to clean the tracks from any location mistakes caused by the tracking algorithm. The motion model for the Kalman filter was based on the spider's position, speed, and acceleration. Its starting position, speed, and acceleration were absolute while all the consequent values were calculated according to the tracked positions considering the inherent Gaussian noise found in the data.

Video recordings allow extensive exploration of the spiders' movement patterns. The spiders' departure paths had a noticeable sinusoidal appearance and the shape of the paths was described by the wavelength and amplitude of the sinus wave. The path wavelength was determined by the distance between the intersection points of the path with a regression line that best fit the path's general direction. The maximal distance of the path from the regression line with in each sinusoidal cycle resulted in the path's amplitudes. The departure path wavelengths and amplitudes were averaged for each exit. PERMANOVA (Permutational ANOVA) was used to determine significant differences in the departures' mean-wavelengths and mean-amplitudes depending on order of departure. The data was first transformed with a fourth root, and as measure of distance a Bray Curtis similarity matrix was used. A type I (sequential) sum of squares with fixed effects sum to zero for mixed terms and unrestricted permutation of raw data was used (maximal number of minimum permutations: 9951).

**Figure 2:**
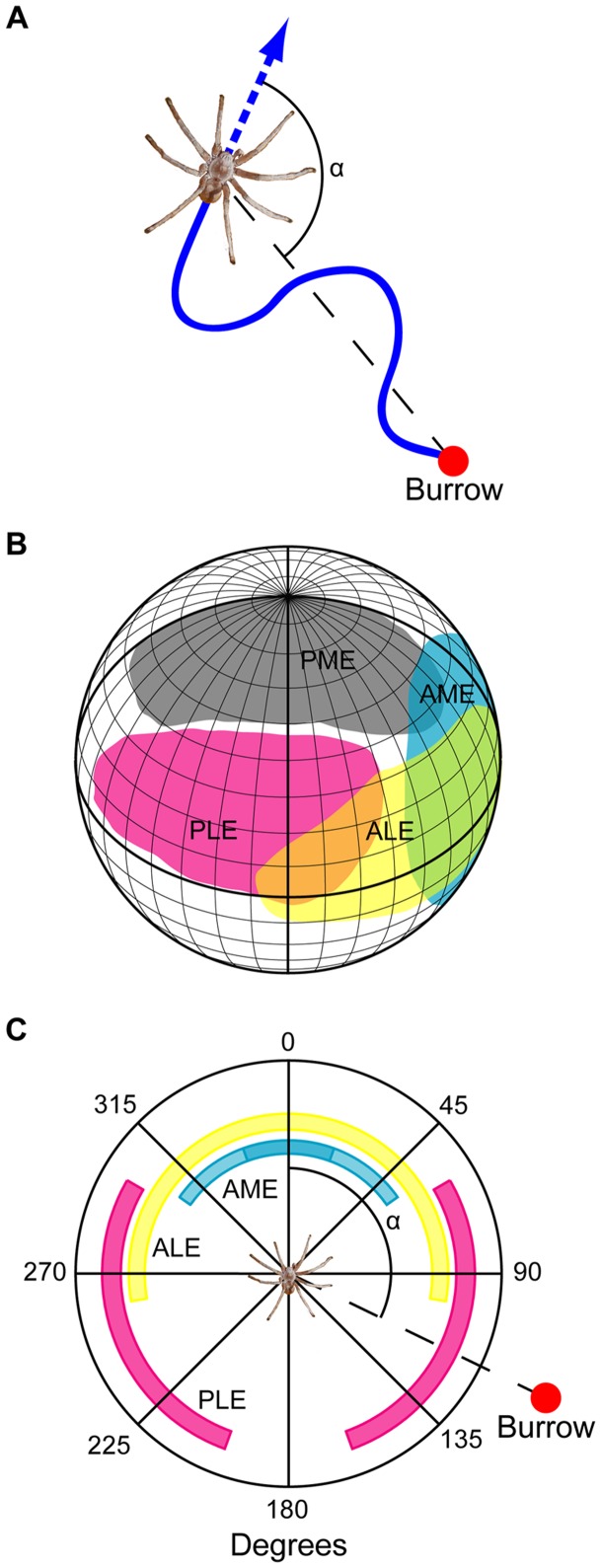
Which eyes are looking back? **A.** The body-to-burrow alignment angle α measured as the angle between the spiders' current walking direction and the direct line to the burrow. **B.** The visual fields of the anterior median eyes (AME) (light blue), anterior lateral eyes (ALE) (yellow), posterior median eyes (PME) (grey), and posterior lateral eyes (PLE) (magenta) on the right side of a *L. arenicola* visualised on a globe viewed from 15° above the horizontal plane (modified from Nørgaard et al. 2008). **C.** A visual field compass composed of the spider's visual fields (except the upwards-looking PME): this can be used to determine which eye(s) look back at the burrow for any given α.

Unlike insects, spiders do not have heads that can move independently of their bodies, but have their eyes as well as their legs attached on the prosoma. Moreover, it is only the anterior median eyes that have variable visual fields due to having moveable retinas – the visual fields of the other six eyes are fixed [30]. It is therefore possible to deduce directly and with high certainty where each of the spider's eyes are looking simply based on the animal's walking direction. This represents a great advantage over comparable insect models, such as bees and ants, where head movements can affect viewing direction significantly [31]. We determined the body-to-burrow alignment angle α as the angle between the spiders' walking direction and the direction to the burrow position (Fig. 2A). The walking direction was measured as the straight line between the spiders' position in consecutive frames of the video recordings. Since the visual fields of each of *L. arenicola's* eyes have previously been established [6] (Fig. 2B) it was possible to determine which eyes were looking in the direction of the burrow position during the departures (Fig. 2C).

We used Generalized Linear Mixed Models (GLMM) with a Gaussian error structure and an identity link function to test for significance. A Markov chain Monte Carlo (MCMC) sampled (10000 iterations) the GLMM to find the P values (all calculations were done in R 2.10.1, libraries: lme4, coda, and languageR) [32]–[34]. This combination was used to investigate how outgoing versus returning excursions as well as the excursion number affected the spider's path length, the time spent on the path, and its mean speed (these variables were box cox transformed for normality). These three variables are naturally highly correlated excluding pauses where the spiders stood still. We therefore chose to test each variable separately. In all our tests, the spider identity was the random factor. An initial investigation showed a strong interaction (P = 0.01) between the two factors, direction and excursion number. To better understand the effect excursion number had on the variables, we tested for significance for each of the directions (incoming and outgoing) separately. The speed with which the spiders distanced themselves from the burrow during the departure was measured as the spiders' direct distance to the burrow as a function of time.

## Results

**Figure 3:**
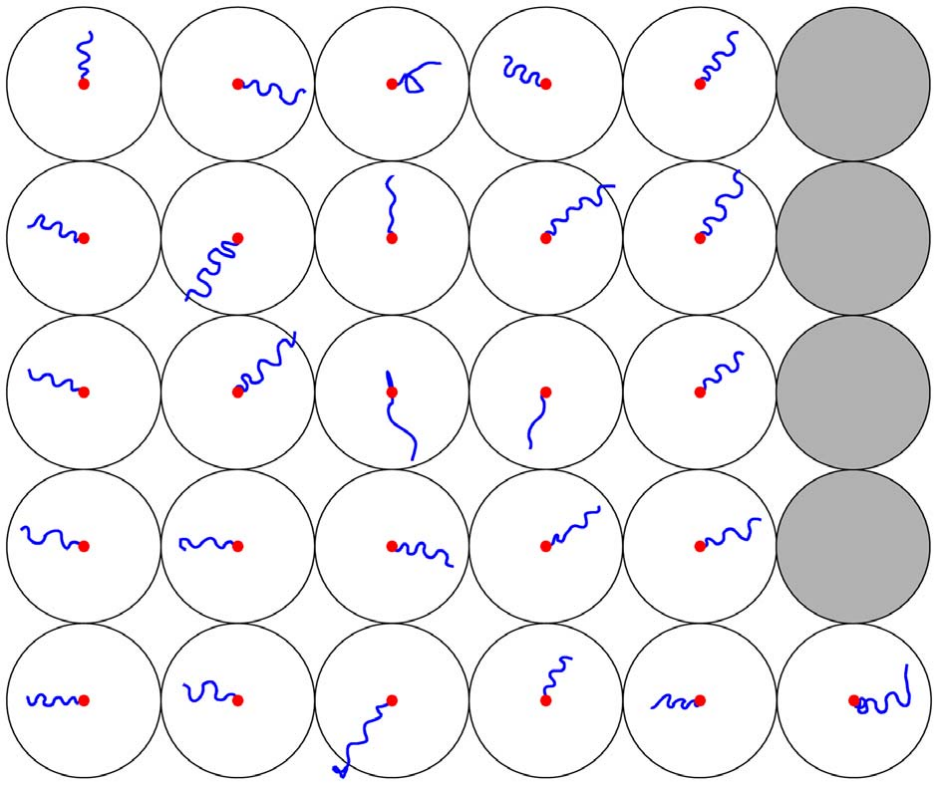
Departures by naïve spiders. Illustrations of the 26 departures recorded from individual male spiders leaving their burrow for the first time in a completely unfamiliar area. The red dots mark the burrow position. The circles represent the 2 m radius circle around the burrow.

**Figure 4:**
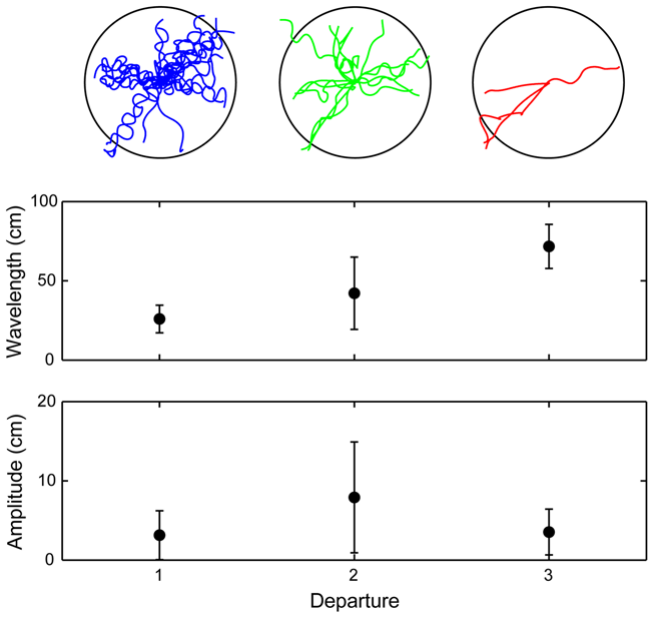
Ontogeny of sinusoidal departure pattern. The x-axis denotes the sequential departure of a spider from its burrow (1^st^: N = 26, 2^nd^: N = 16, and 3^rd^: N = 4). Departure paths are shown in the top pane. The circles represent the 2 m radius circle around the burrow. In the middle pane are the mean wavelengths of the sinusoidal paths. The amplitudes are shown in the bottom pane (y-axis is in cm). Error bars are standard deviation. While the amplitudes were not significantly different between departures, the wavelengths were (PERMANOVA, *P* = 0.0003).

First departures from naïve spiders were recorded on 26 nights (N = 26) and while two consecutive excursions per night were frequent (N = 16), three were rare (N = 4). Most first departure paths had a distinct sinusoidal shape (Fig. 3). When possible, visual observations of the spiders' foot prints in the sand revealed that the sinusoidal pattern rapidly ceased beyond the camera's field of view (2 meters from the burrow). The sinusoidal shape became noticeably less and less prominent on consecutive departures and statistical tests proved that the mean wavelengths and mean amplitudes of the sinusoids were significantly different between the 1^st^, 2^nd^, and 3^rd^ excursions (PERMANOVA, *P* = 0.0003) (Fig. 4). As consecutive departures became straighter and straighter the mean wavelength became longer (Fig. 4). The mean amplitudes, however, did not exhibit this trend.

**Figure 5:**
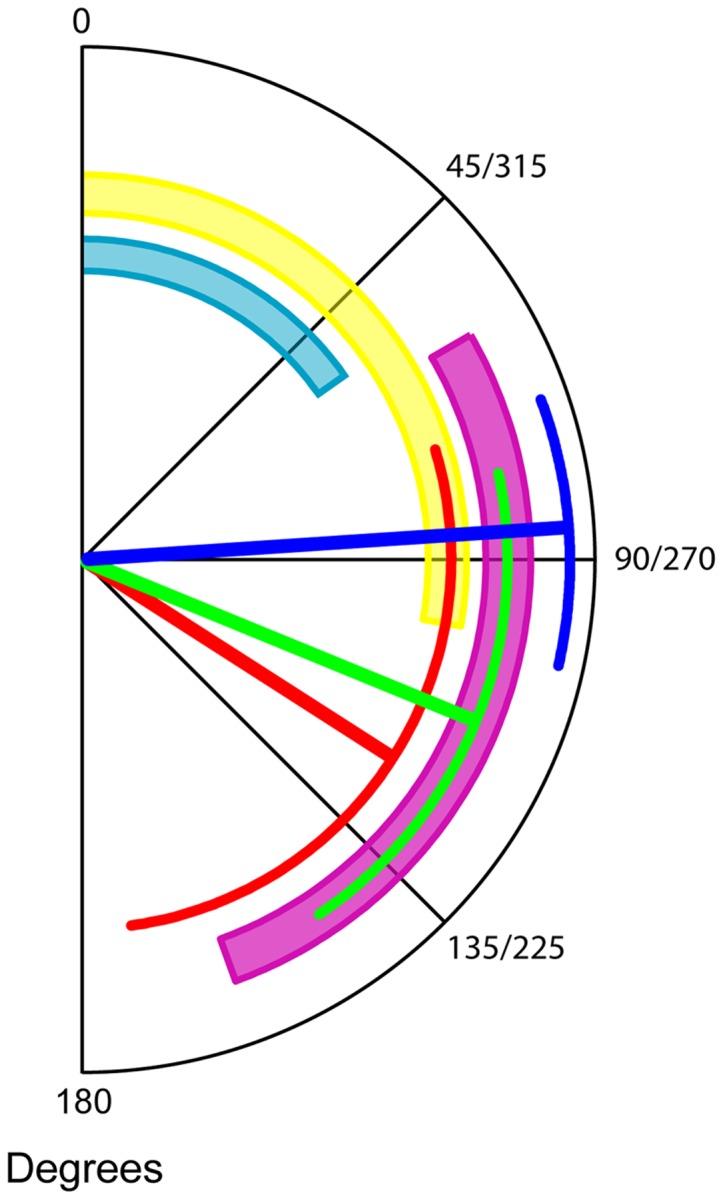
Effect of departure sequence on body-to-burrow alignment angle α. Mean α and 95% confidence intervals for 1^st^ (α  =  blue, N = 26), 2^nd^ (α  =  green, N = 16), and 3^rd^ (α  =  red, N = 4) departures on single nights pooled for left and right side depicted on a compass with the visual fields of the AM eyes (light blue), AL eyes (yellow) and PL eyes (magenta).

The body-to-burrow alignment angle α (the angle between the burrow and the spider's walking direction) was calculated along all paths. Using this angle, we determined which of the spiders' eyes were looking back at the burrow during departures. During the first departures the spiders appear to be keeping the position of the burrow in the overlapping zone of the visual fields of the lateral eyes. Thus for the majority of the duration of the departures the ipsilateral anterior and posterior lateral eyes are both used to record the burrow's position (Fig. 5). In consecutive departures the involvement of the anterior lateral eyes appears to be less important and finally only the posterior lateral eyes are looking back towards the burrow position (Fig. 5).

**Figure 6:**
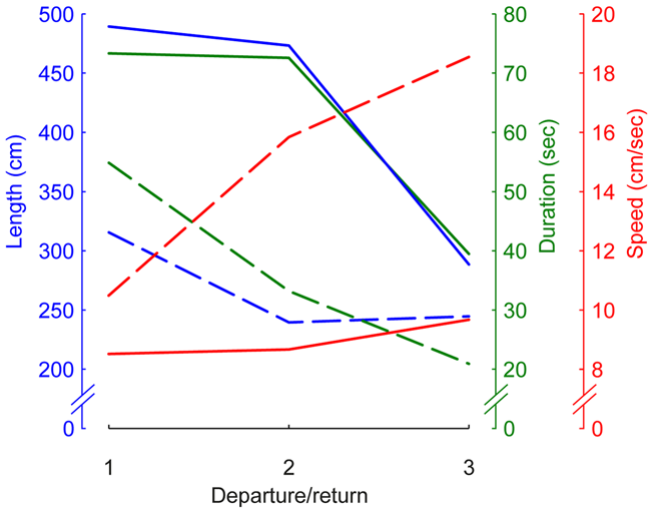
Departures compared to returns. Mean departure (dashed lines) and return (solid lines) path length (cm), duration (s), and speed (cm/s) as a function of departure or return number on single nights (see Table 1 for descriptive statistics).

**Figure 7:**
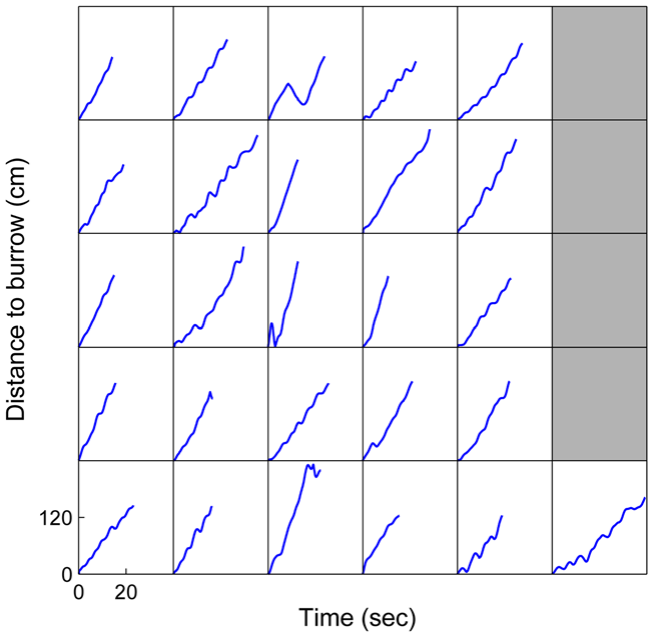
Constant rate of departure. The direct distance to the burrow as a function of time in individual spiders reveals that the spiders move away from their burrow at a constant rate independent of the shape of the sinusoidal movement pattern they perform.

The overall average spider walking speed was 9.6±25.2 cm/sec (mean and variance of a fitted Rayleigh distribution to measured speeds per video frame for all recorded footages (∼53 minutes) R2>0.99). We found that the spider's path length, the time spent on the path, and its mean speed were significantly affected by excursion number for outgoing trips (Table 1 and Fig. 6, dashed lines), where each consecutive exit was shorter (P = 0.0004), took less time (P<0.0001), and was faster (P<0.0001). The same was not true for incoming trips (Table 1 and Fig. 6, solid lines), where excursion number did not significantly affect the path length (P = 0.4717), time spent (P = 0.1516), or speed (P = 0.3932). A test for significance between outgoing and returning excursions showed a very significant difference (P<0.0001 for all three variables). Returning trips were longer, took more time, and were slower than outgoing excursions. Measurements of the speed with which departing spiders distance themselves from their burrows (i.e. straight distance between spider and burrow divided by time) revealed that although there are individual differences they all increase the distance to the burrow at a roughly constant rate (Fig. 7).

**Table 1:** Descriptive statistics for excursion departures and returns.

		Path length (cm)		Duration (s)		Speed (cm/s)	
Direction	Excursion #	mean	s.d.	mean	s.d.	mean	s.d.
Departure	1	315.5	90.3	54.9	39.4	10.5	3.1
	2	239.5	99.1	33.2	36.9	15.8	4.9
	3	244.7	64.5	20.9	5.6	18.5	4.3
Return	1	489.4	321.4	73.3	43.7	8.5	1.8
	2	473.3	220.5	72.6	40.1	8.7	2.2
	3	288.5	70.6	39.5	12.1	9.7	2.2

## Discussion

Vision is the primary sensory modality used by *L. arenicola* when homing [6]. The spiders do not use olfaction or any other non-visual sensory cues when locating their burrows [10]. In landmark-rich areas they can even return to their burrows after passive displacements, showing that they utilise the visual scenery to pin-point the goal location [10]. The sinusoidal departure paths we observed are therefore likely to be learning walks where the spiders familiarise themselves with features of the visual surroundings that uniquely identify the burrow's location. Similar to what is seen in homing insects, the sinusoidal shape of the spiders' departure path is confined to the area close to the goal and the movement patterns are especially pronounced in naïve individuals leaving the goal for the first time [20]. The sinusoidal shape of departure paths rapidly becomes less pronounced during consecutive departures. This suggests that the spiders' are capable of visual learning and memory [28]. A comparable pattern change is also seen in the learning flights and walks of the above-mentioned hymenopterans where the specialised movement patterns also become less and less pronounced as the insect gains experience [20].

Solitary wasps learning flights consist of arcs increasing in size, height above ground, and flight velocity (ground speed) as the wasp moves away from its nest [19]. In this way the wasp keeps the nest at a fixed retinal position and it maintains a constant angular velocity in relation to the nest [19]. This does not appear to be a strategy used by the spider where the amplitude of the sinusoidal departures does not increase as it moves away from the burrow. Social wasps have so-called inspection points at the end of the learning flight arcs [24]. These wasps thereby view the goal from vantage points along lines extending from the goal and it is likely that they acquire information about the goal position at the inspection points [24]. The spiders do not appear to have specific inspection points along the sinusoidal path but the behaviour is also not clearly defined in honey bees or solitary wasps [24].

The spiders' departures, but not the returns, became shorter and faster with experience (Fig. 6). This indicates that the spiders may learn most during the first departure and do not add much, but rather merely refresh their memory during the following departures. The many similarities between the learning flights and walks of diurnal insects and the departure walks of the spider support our theory that these striking stereotyped movement patterns are learning walks.

Bees and wasps – including nocturnal species – face towards their nest during learning flights, as do Namib Desert ants when pausing during their rotational movements [3], [21], [35]. The spiders, however, do not turn around and look back, but rather take sideways looks at the burrow position with their lateral eyes (Fig. 5). Recent reports have shown that the lateral eyes in spiders can perform advanced functions [36]–[38]. This may also be the case in *L. arenicola*. The spiders have eyes on both sides of the prosoma and could in principle scan the panoramic view away from the burrow during their sinusoidal departures. The burrow entrance is closed with a lid of silk and sand grains making it nearly impossible to see, even for a human observer in daylight [8]. It is therefore striking that the camouflaged burrow entrance, although imperceptible to the spider in the dark, is so accurately kept in the overlapping part of the lateral eyes' visual fields during the first departure. Why spiders would need to keep their burrow-entrance in this overlapping visual field zone is still uncertain, but the key to understanding this behaviour can most likely be found in the neural architecture of the spiders' visual system. In the Wolf spider *Lycosa tarantula* there are neural connections between the visual pathways of the anterior and posterior lateral eyes [39] and this could also be the case in *L. arenicola*. The use of two eyes rather than just one could theoretically provide the spiders with higher sensitivity or greater spatial acuity in the dark. Whatever role the overlapping visual fields have in these spiders, it is interesting to note that bees and ants likewise have overlapping zones in the frontal part of their visual fields that point towards their goal during their learning flights and walks [3], [40]. Unless the spiders use landmark cues very near the burrow entrance, their ability to keep the goal at a specific retinal position is furthermore an indication of an underlying path integration system [21]. During the departures the spiders' direct distance to the burrow increased at a steady rate. This would lead to a steady change in any motion parallax experienced by the spiders and would facilitate the disentanglement of the three-dimensional layout of landscape features. This therefore suggests that they use motion parallax for depth perception. Motion parallax as a method for obtaining three-dimensional information is widely used in many animals, including bees and wasps [41]–[44].

Analysing observations of the spiders' behaviour does not hold the same power as direct experimental manipulations. However, the spiders' dependence on vision, and especially their inability to use olfaction when navigating [10], makes it unlikely that their behaviour is based on other sensory modalities [6]. Numerous earlier recordings of complete *L. arenicola* excursions did not show the sinusoidal patterns at the initial part of a departure [8], [12], [13]. However, these paths were all recorded from spiders that had prior experience with their surroundings. Our discovery of sinusoidal departure patterns in naïve individuals has therefore uncovered a hitherto unknown feature of this spider's nocturnal navigation.

When leaving an unfamiliar goal locality homing insects display stereotypic movement patterns associated with visual learning that probably form the base of some type of snapshot matching [3], [25]. Unless the theoretical limitations believed to impede long-distance ideothetic navigation by proprioceptive input only (e.g. from the spiders' lyriforme organs) are far too strict [45]–[48], our observations strongly suggests that *L. arenicola*, despite being nocturnal, likewise uses a visual homing strategy based on snapshot matching. Future work will determine whether this is indeed the case.
